# A Novel Microwave Resonant Sensor for Measuring Cancer Cell Line Aggressiveness

**DOI:** 10.3390/s22124383

**Published:** 2022-06-09

**Authors:** Livio D’Alvia, Serena Carraro, Barbara Peruzzi, Enrica Urciuoli, Luigi Palla, Zaccaria Del Prete, Emanuele Rizzuto

**Affiliations:** 1 Department of Mechanical and Aerospace Engineering, Sapienza University of Rome, 00184 Rome, Italy; livio.dalvia@uniroma1.it (L.D.); serena.carraro@uniroma1.it (S.C.); zaccaria.delprete@uniroma1.it (Z.D.P.); 2 Multifactorial Disease and Complex Phenotype Research Area, Bambino Gesù Children’s Hospital, IRCCS, 00146 Rome, Italy; barbara.peruzzi@opbg.net (B.P.); enrica.urciuoli@opbg.net (E.U.); 3 Department of Public Health and Infectious Diseases, University of Rome La Sapienza, 00185 Rome, Italy; luigi.palla@uniroma1.it

**Keywords:** measurement of dielectric properties, biosensor, microwaves, noninvasive measurements, cancer cell lines, cancer aggressiveness, osteosarcoma, breast cancer

## Abstract

The measurement of biological tissues’ dielectric properties plays a crucial role in determining the state of health, and recent studies have reported microwave biosensing to be an innovative method with great potential in this field. Research has been conducted from the tissue level to the cellular level but, to date, cellular adhesion has never been considered. In addition, conventional systems for diagnosing tumor aggressiveness, such as a biopsy, are rather expensive and invasive. Here, we propose a novel microwave approach for biosensing adherent cancer cells with different malignancy degrees. A circular patch resonator was designed adjusting its structure to a standard Petri dish and a network analyzer was employed. Then, the resonator was realized and used to test two groups of different cancer cell lines, based on various tumor types and aggressiveness: low- and high-aggressive osteosarcoma cell lines (SaOS-2 and 143B, respectively), and low- and high-aggressive breast cancer cell lines (MCF-7 and MDA-MB-231, respectively). The experimental results showed that the sensitivity of the sensor was high, in particular when measuring the resonant frequency. Finally, the sensor showed a good ability to distinguish low-metastatic and high-metastatic cells, paving the way to the development of more complex measurement systems for noninvasive tissue diagnosis.

## 1. Introduction

Recently, techniques for measuring materials’ dielectric properties have become a pivotal issue due to their impact on scientific research, for example, for industrial, communication, and environmental applications [1,2,3,4,5]. Recently, various systems based on microwave biosensing have achieved significant benefits in healthcare and biomedical fields due to their minimal invasiveness, high versatility, and sensitivity [6,7,8].

Microwave sensors offer the possibility of measuring living tissue’s dielectric properties through noninvasive scattering parameters [9]. Changes in these factors, which are strictly related to the resonant frequency of the probe, could be crucial for monitoring or diagnosing possible pathological states [10,11]. For instance, different sensing devices have been used for monitoring vital signs (e.g., heart rate, blood pressure, and blood glucose) [12,13], as well as for the detection of specific biomolecules [14], or even diseases such as cancer and Parkinson disease [15,16,17]. For example, Chen et al. proposed a capacitive biosensor modified with silica-alumina to detect glucose levels and to provide a continuous monitoring of diabetes [18]. Others have shown that the dielectric properties of cancer tissues changed with increasing water content [19,20], or that dielectric alterations were well correlated with the carcinogenicity of thyroid nodules [21]. Moreover, Lazebnik et al. investigated the dielectric permittivity changes in breast tissue when affected by malignancy as compared with healthy breast tissue, using a sensor in the range of 0.5–20 GHz [22]. Thus, changes in dielectric properties of biological tissues could be the turning point for monitoring physiological parameters and preventing the possible onset of diseases or for pathology detection.

Early tumor detection is a critical part of cancer research for reducing the mortality rates of cancers. However, due to the complex and various signaling pathways involved in cancer development, there is currently a lack of knowledge on tumor behavior [23,24,25]. Generally, the diagnosis is based on the degree of the tumor, which is determined by cancer cell properties during the tumor lesion development. For example, a high degree of malignancy indicates the presence of aggressive cancer cells that proliferate and spread more quickly than non-aggressive cells [26,27,28]. Although several conventional technologies for cancer detection exist, such as a biopsy, they are quite expensive and invasive [29,30]. Thus, microwave biosensors could represent a substitute or additional approach for noninvasive diagnostic detection of various diseases at their early stage, especially for cancers. Increased interest in the microwave methodology has led to satisfying the demanding requirements for sensors, such as high sensitivity and noninvasive detection, starting from the cellular level [31,32,33]. These cell-based systems have been used to measure cellular dielectric properties in a controlled micro-environment under *in vitro* conditions, paving the way for *in vivo* diagnostic applications.

In this context, here, we propose a microwave-based biosensor for the noninvasive detection of cancer cell types with varying degrees of aggressiveness. The resonator was initially designed in the CST Microwave Studio software to properly match the structure of the Petri cell dish surface. This was followed by simulation analyses which were computed to study its response, testing the proposed biosensor in air and with a common basal medium for cellular growth. Finally, the sensor was realized and tested to discriminate between two pairs of adherent cancer cell lines with varying degrees of aggressiveness.

## 2. Materials and Methods

### 2.1. Sensor Design and Realization

**Figure 1:**
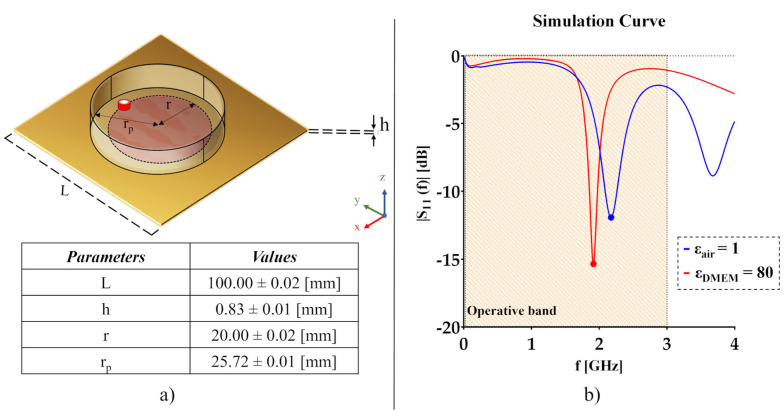
(**a**) Design and dimensions of the circular biosensor with the Petri dish; (**b**) Simulation response designed probe in the air (*ε_air_* = 1.00) and in cell culture medium (*ε_DMEM_* = 80.00), highlighting the operative frequency range of the VNA (0.001–3.000 GHz).

In this work, we proposed a circular patch biosensor to detect four cancer cell lines with different origins and varying degrees of aggressiveness in adhesion on a standard Petri (Falcon^®^, 60 x 15 mm) dish surface. To this end, we modified a prototype previously realized [34], by parametrizing the probe dimension and connector positioning. Simulations were performed with the CST Microwave Studio software to increase its sensitivity as much as possible but taking into consideration the operational range of the network analyzer employed in the subsequent experiments. The final configuration of the sensor and the dimensional values are reported in Figure 1a. The structure is composed of a square grounded dielectric layer (*L* = 100.00 mm) comprising a circular dielectric patch element at the center (*r* = 20.00 mm). The sensor was designed on a 0.76 mm Rogers RO4835 dielectric layer with a dielectric constant (*ε_sub_*) and a loss tangent (*tan_sub_ (δ)*) of 3.66 and 0.0031, respectively. The total thickness of the probe was set to *h* = 0.83 mm. The circular shape of the patch was chosen to guarantee the maximum symmetry below the circular Petri dish surface. It should be noted that the patch radius and the position of the SubMiniature version A (SMA) connector were crucial parameters for the optimal working of the probe, considering their strict relation with the operative resonant frequency. Therefore, we chose a patch radius (*r* = 20.00 mm) smaller than that of the Petri dish (*r_p_* = 25.72 mm), and the coaxial waveguide connector was soldered at the border of the patch for a good impedance. Since we expected to use this sensor in an operating frequency range of 0.001–3.000 GHz, these choices represented the optimal compromise between the resonant frequency and sensor sensitivity within the above reported operating microwave range.

The working principle of the probe consists of the electromagnetic field propagation through the SMA connector, thus, exciting the sensor structure and the material under test (MUT) due to resonance. The reflection coefficient-based method was chosen to evaluate the sensor’s operation by studying the minimum of the scattering parameter |*S*_11_ *(f)*| corresponding with the associated resonant frequency (*f_r_*). The operating frequency was computed through the following equation [35]:

(1) $$f_{r}=\frac{k_{1,1}\cdot c}{2\pi r_{e}\sqrt{\varepsilon_{e}}}$$

By setting the first zero of the derivative Bessel function of order one (*k*_1,1_*)* at 1.84 and the effective radius (*r_e_*) of the patch at 20.36 mm, and by varying the effective dielectric permittivity (*ε_e_*), of note, the resulting *r_e_* is slightly larger than the designed one (*r* = 20.00 mm) for the influence of the fringing field at the edges. Since the effective dielectric constant *ε_e_* is strictly related to the radiated material, in simulation analyses we calculated this parameter considering two conditions, one with only the contribution of the probe dielectric substrate in the air (*ε_e___sub_* = 3.61) and one with the presence of a MUT, namely the Dulbecco’s modified Eagle medium (DMEM) for cell growth (*ε_e___DMEM_* = 5.04). Based on the literature, the relative permittivity was set at 1.00 and 80.00 for the air and the medium, respectively [36,37]. The theoretical results pointed out an expected frequency shift due to the presence of MUT towards lower frequencies, from 2.270 GHz in the air to 1.923 GHz with the DMEM medium. 

Figure 1b shows a representative simulation of the biosensor response, testing the prototype without (*ε_air_* = 1.00) and with the DMEM (*ε_DMEM_* = 80.00). The tests were performed in the CST Microwave Studio software and the simulations showed that the sensor produced a different output for different MUT. Considering the operative frequency band of 0.001–3.000 GHz, both curves differed from each other in the minimum of reflection coefficient (*|S_11_ (f)|*_air_ = −11.93 dB and *|S*_11_ *(f)|_DMEM_* = −15.35 dB), as well as in resonant frequency (*f_r_DMEM_* = 1.916 GHz and *f_r_air_* = 2.175 GHz). These preliminary results suggested a possible two-fold sensitivity of the biosensor in terms of signal *min|S_11_ (f)|* and resonant frequency shift. 

Of note, the simulation results agreed with the theoretical results in terms of resonant frequency with a relative error of 4.2% in air, i.e., without any MUT.

**Figure 2:**
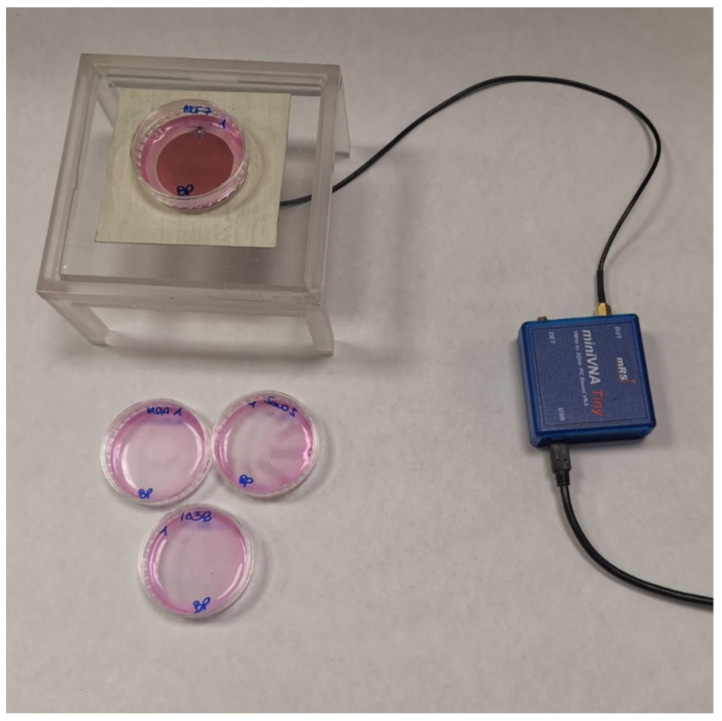
The experimental set-up comprises the circular biosensor, the network analyzer, and the Petri dish.

Figure 2 shows the set-up employed for the experiments with cultured cancer cell lines. The circular probe was realized using the CNC milling machine 3020T 3D (MTechnic^®^, Taiwan), following the previous design specifications. The SMA waveguide connected the probe with a vector network analyzer MiniVNA-TINY for measuring scattering parameters in the operating frequency range of 0.001–3.000 GHz. The system also comprised a USB serial port, supported by the software VNA/J [38]. A plexiglass support was realized ad hoc to guarantee proper positioning of the Petri dishes over the probe.

### 2.2. Cell Culture and Sample Preparation

**Figure 3:**
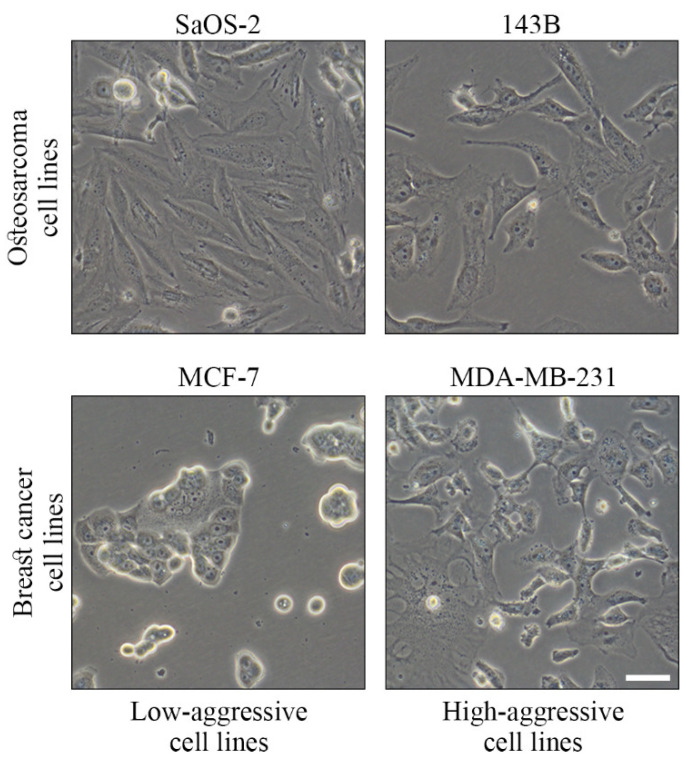
Representative images (40X) of the cancer cell lines on the day of the experiment, 24 h after plating in a 60 mm Petri dish. Bar, 100 µm.

For this work, low-aggressive osteoblast-like osteosarcoma SaOS-2 cells [39], high-aggressive lung-tropic metastatic osteosarcoma 143B cells [40], low-aggressive breast cancer MCF7 cells [41] and high-aggressive bone-tropic breast cancer MDA-MB-231 cells [42] were employed to assess the capability of the proposed microwave resonant sensor to distinguish cancer cell aggressiveness. Pediatric human osteosarcoma cell line SaOS-2 (HTL01001), human breast adenocarcinoma cell lines MCF7 (HTL95021) and MDA-MB-231 (HTL99004) were purchased from Banca Biologica and Cell Factory (IRCCS Azienda Ospedaliera Universitaria San Martino-IST, Genova, Italy), while the pediatric osteosarcoma cell line 143B (CRL-8303) was purchased from the American Type Culture Collection (Manassas, VA, USA). All cell types were grown in Dulbecco’s modified Eagle’s medium (DMEM, Euroclone) supplemented with 10% fetal bovine serum (FBS, Corning), 100 units/mL penicillin/streptomycin (Euroclone) and maintained at 37 °C in 5% CO_2_. After a series of preliminary experiments, we decided to test all the cell lines 24 h after plating, which was the time of optimal compromise between good cell adhesion and cell confluence. To this end, since the four cell lines have different growth rates, for each experiment, a specific number of cells (SaOS-2, 5 × 10^5^ cells; 143B, 3.6 × 10^5^ cells; MCF7, 7.5 × 10^5^ cells; and MDA-MB-231, 6.4 × 10^5^ cells) was plated at the “time-point 0” in 1.5 mL of DMEM in standard 60 mm Petri dishes in order to achieve a comparable number of cells (7.5 × 10^5^ < x < 9 × 10^5^) at the time of the measurements of the dielectric properties, confirmed by cell count at the end of the test. Representative images of the tested cell lines on the day of the experiments are shown in Figure 3. Images were captured using an inverted microscope (Olympus IX71).

### 2.3. Experimental Procedure

To evaluate the sensor capability to distinguish cell types with varying degrees of aggressiveness, we performed cellular experiments involving two pairs of cancer cell lines, namely low-aggressive SaOS-2 and high-aggressive 143B osteosarcoma cells, and low-aggressive MCF7 and high-aggressive MDA-MB-231 breast cancer cell lines. In addition, DMEM was also tested as a control. The medium volume was set equal to 1.5 mL in all the conditions and was prepared by pouring the medium into each Petri dish with successive fillings of 750 μL with a precision pipette (Eppendorf^®^ Research^®^ plus pipette 100–1000 μL), with an uncertainty lower than 0.25% for both systematic and random errors. Eight samples were tested for each of the five conditions (four cell lines and one control), and the experiments were performed on three different days. For each sample, one single measurement was performed with the use of the above-described set-up.

Of note, four small markers were drawn to increase reproducibility in the placement of the dishes. Nevertheless, a series of preliminary tests was carried out to evaluate possible errors due to dish positioning. Three Petri dishes containing DMEM were tested with 10 repeated measurements still on the probe, and 10 times being removed and replaced before each measurement. The results showed only a modest increase in the variance of all the measured parameters (e.g., +0.15 MHz on average for the frequency) when removing the dishes before each measurement and then still tested on the probe.

### 2.4. Data Elaboration Process

For each of the eight samples of the five materials under test (MUTs), we acquired and analyzed the reflection coefficient *|S_11_ (f)|.* To increase the resolution of the acquired data as much as possible, we imposed a sampling frequency of 0.5 MHz in the range from 1900.0 to 2600.0 MHz. The reflection coefficient was fitted to a Lorentzian curve (Equation (2)) using a nonlinear least squares method [43]:

(2) $$L(x)=\frac{A}{1+{\left(\frac{x-x_{c}}{x_{h}-x_{l}}\right)}^{2}}$$

where *A* is the minimum of the curve; *x_c_* is the center at the minimum of the Lorentz curve; and *x_l_* and *x_h_* are the lower and upper bounds placed at the half-height of the curve, respectively.

By the fitting, we assessed the minimum of *|S_11_ (f)|*, the correspondent resonant frequency *f_r_*, evaluated at *x_c_*, the full width at half maximum (*FWHM*) and the interpolation error in terms of square error. At this point, it should be noted that we decided to employ the *FWHM* instead of the Q factor [44,45,46], since this parameter is more coherent with the Lorentzian fitting we performed [43].

Data analysis was performed with GraphPad Prism 6.0.

### 2.5. Statistics

For all the measured parameters, the values are expressed as mean ± SD. The coefficient of variation (CV) was computed as the ratio between the SD and the mean value, and expressed as a percentage. The overall differences in the tumor cell lines, consideringalso the day of the experiment as an additional factor on, were assessed by multivariate analysis of variance (MANOVA) based on Wilk’s Lambda test statistics, including all the parameters obtained from the reflection coefficient, namely *min|S_11_ (f)|*, *f_r_*, and *FWHM*; the MANOVA was also weighted by the sum of square residuals (fitting error) obtained by each Lorentzian curve interpolation procedure. The univariate differences in *min|S_11_ (f)|, f_r_*, and *FWHM*, respectively, between tumor cell lines and between days were analyzed by two-way analysis of variance (ANOVA) weighted by the fitting error obtained for each fitting procedure, followed by multiple comparisons, adjusted by Bonferroni correction.. The statistical analysis was performed with Stata 17, and the differences were considered to be significant when the *p*-value was lower than 0.05. 

## 3. Results

In the present work, two pairs of cancer cell lines with varying degrees of aggressiveness were tested *in vitro* for their dielectric properties using the proposed sensor. 

Table 1 summarizes the results obtained for the three measured parameters in terms of mean value and standard deviation (SD), including the fitting error of the elaborated Lorentzian curves. Interestingly, the coefficient of variation (CV) computed for the pure medium for each of the three parameters derived from the Lorentz fitting showed an acceptablevariability (i.e. CV < 10%) in the *FWHM* (CV = 6.6%) and *min|S_11_ (f)|* (CV = 3.3%), and an extremely low variability in the resonant frequency (CV = 0.02%). In addition, the CV computed for the resonant frequency for the four cell lines resulted almost equal or even lower than that in the DMEM, while the CV computed for the cell lines for the two other parameters resulted higher than in the control medium. 

Table 2 shows the results obtained with MANOVA comparing mean values according to the two factors, tumor and days, for the three dependent variables. The results showed a statistically significant (*p* < 0.0001) difference in factors for the joint variables. This outcome indicated that either tumor or days (or both) were significant for at least one of the dependent variables, highlighting that the two factors overall influenced the measurements.

To determine whether the specific dependent variables were significantly influenced by the two factors, cell line and days, we employed the two-way ANOVA test considering each of the three variables of interest separately, and the results are presented in Table 3 with the effect estimates (and their statistical tests) of the individual factor category for each dependent variable. The results yielded a significant (*p* < 0.0001) influence of the day factor on *min|S_11_ (f)|* and *FHWM*, highlighting that the experimental day affected cell measurements. On the contrary, the frequency variable was significantly (*p* < 0.0001) affected by the cell line factor but not by the day factor. This in-depth analysis revealed that frequency represents the optimal parameter to investigate differences in cells’ dielectric properties, being also robust to changes in the experimental day. 

**Figure 4:**
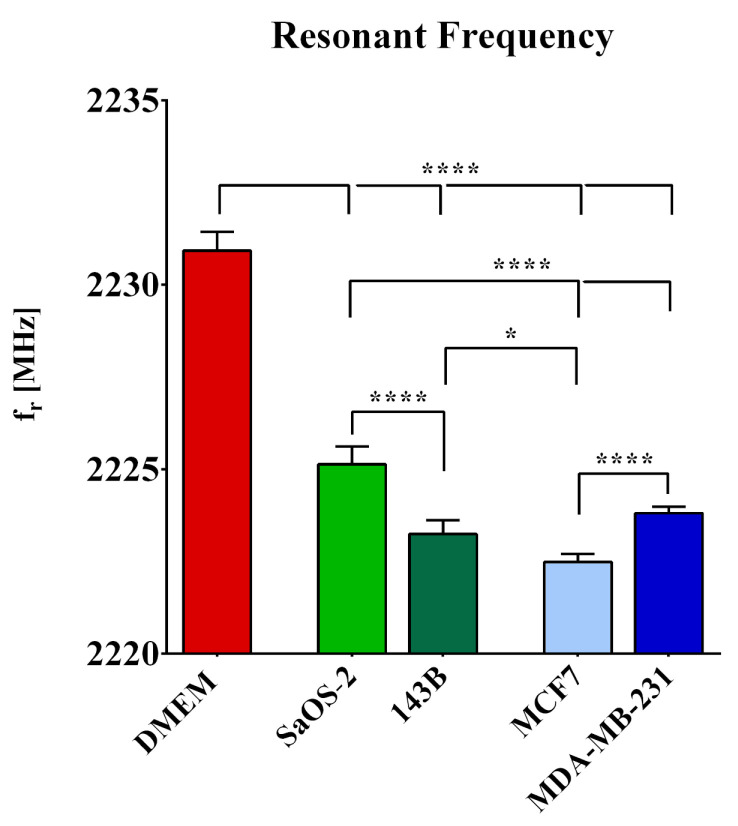
Resonant frequency values computed for pure DMEM, and the four tested cell lines: low-aggressive SaOS-2 and high-aggressive 143B osteosarcoma cell lines, and low-aggressive MCF7 and high-aggressive MDA-MB-231 breast cancer cell lines. *n* = 8. *, *p*< 0.05 and ****, *p* < 0.0001.

Figure 4 shows the frequency values for all the tested groups. ANOVA returned a significant influence of cell type on the resonant frequency. Interestingly, post hoc pairwise comparisons with Bonferroni correction returned a highly significant (*p* < 0.0001) difference between the pure DMEM and each of the tested cell lines, showing that the sensor is capable of detecting adherent cells with respect to medium only, with the resonant frequency of the medium containing cancer cells being consistently lower than that of pure DMEM. 

With regard to the difference in aggressiveness within tumors from the same tissue of origin, post hoc tests revealed a highly significant (*p* < 0.0001) difference between SaOS-2 and 143B cell lines and between MCF-7 and MDA-MB-231 cell lines, showing the potential ability of the sensor to discriminate cell aggressiveness. Interestingly, this was obtained even if the increase in aggressiveness was associated with a different behavior in the two cancer cell types. In particular, high-aggressive 143B osteosarcoma cells showed a decrease in the resonant frequency, while high-aggressive breast cancer cells showed an increase in *f_r_*. This outcome highlighted the high sensitivity of the biosensor in terms of frequency. 

Finally, post hoc tests showed statistically significant differences in the frequency value of high-aggressive cells as compared with low-aggressive cells, even for cells from different tumor types, namely 143B osteosarcoma cells versus MCF7 breast cells and MDA-MB-231 breast cells versus SaOS-2 osteosarcoma cells. 

**Figure 5:**
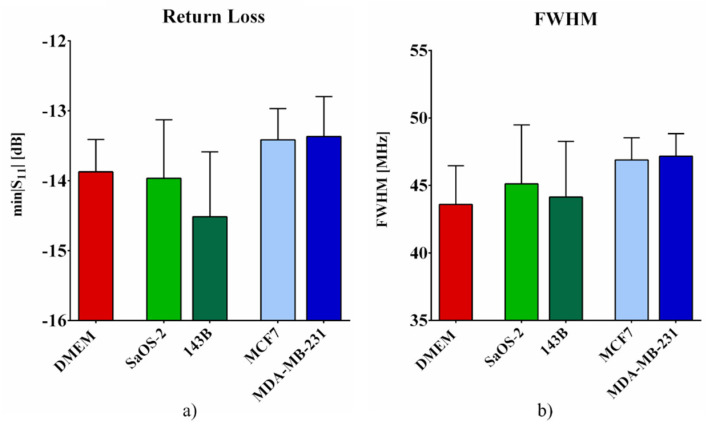
*Min|S_11_ (f)|* (**a**) and *FWHM* (**b**) computed for pure DMEM, and the four tested cell lines: low-aggressive SaOS-2 and high-aggressive 143B osteosarcoma cell lines, and low-aggressive MCF7 and high-aggressive MDA-MB-231 breast cancer cell lines. *n* = 8.

Figure 5 shows the *min|S_11_ (f)|* and *FWHM* values for all the tested groups. The results also reflect the fact that these two parameters were affected by a higher measurement variance than that reported for the resonant frequency. 

## 4. Discussion

The aim of this work was to design a new sensor to assess the aggressiveness of cultured cancer cell lines, i.e., when cell–cell interactions and cell–substrate adhesion play a crucial role in their growth [47]. To this aim, we proposed a novel sensor based on the measurement of the specimen’s dielectric properties through the microwave methodology, an approach widely used in industrial [48] and, more recently, in biomedical [49] applications. This approach guarantees the possibility to develop low invasive, cost-effective, and easy-to-use devices. The proposed device was realized and employed to test four different cell lines in comparison to culture medium, and three parameters were chosen to characterize the tested groups: the resonant frequency *(f_r_)*, the minimum of the scattering parameter *|S*_11_ *(f)|* in correspondence with the associated resonant frequency, and the full width at half maximum *(FWHM)*. It should be noted that the biological samples cannot be considered to be reproducible samples, and the culture medium was the only material that could be employed to test measurement repeatability. The experimental results obtained with a culture medium showed an acceptable variability for all the tested parameters, with coefficient of variation (CV) values always lower than 7%, on average. However, the coefficient of variation value computed for the resonant frequency was about two orders of magnitude lower, highlighting a higher accuracy in measuring this parameter. At the same time, the resonant frequency was more robust to the changes in material under test than the *|S*_11_ *(f)|* and *FWHM*, since its CV values were almost constant for all the tested cell lines. These outcomes are in accordance with the theory underlying the probe design, in which the resonant frequency is the main parameter characterizing the probe response, followed by the *FHWM* and *min|S_11_ (f)|.* Finally, the resonant frequency was also more robust to potential confounding of external factors, such as the day of the experiment. As a matter of fact, the experimental results showed a significant frequency shift among each of the tested cell lines and the culture medium, and between low-aggressive and high-aggressive osteosarcoma cell lines and between low-aggressive and high-aggressive breast cancer cell lines. Interestingly, a significant frequency shift was also reported between low-aggressive osteosarcoma and high-aggressive breast cancer cell lines and between low-aggressive breast cancer and high-aggressive osteosarcoma cell lines, highlighting the ability of the proposed sensor to discriminate cancer cell line aggressiveness regardless of cancer type. Indeed, even though this result could not be generalized to other malignant cell types, further studies could be conducted to determine whether specific changes occur in highly aggressive cells that directly affect their dielectric properties. In accordance with the accuracy and robustness outcomes, the experimental results showed that the measurements of *min|S*_11_ *(f)|* and *FWHM* did not allow for a precise detection of cancer cell lines, either with respect to the medium, or among the different cells.

As previously stated, in this work, we focused on adherent cells, to consider the cell-to-cell and cell-to-substrate interactions, while others have focused on the detection of cells in suspension in their culture medium [50] even a single flowing cell [32], or low-concentration cells outside a culture medium [51]. However, in general, the results pointed out electromagnetic signature differences between the tested cell lines, highlighting, in particular, that cells’ dielectric properties are highly related to cells’ concentrations. Indeed, since the four cell lines tested in our work have different growth rates according to their aggressiveness, in our work, we strictly monitored their number to guarantee very similar conditions on the experimental day. Of note, in this work, we decided to test cell cultures in a condition which resulted in an optimal compromise between good cell adhesion and cell confluence, but other experiments could be performed in “subconfluent” and “over-confluent” conditions to evaluate whether the differences in the resonant frequency among the cell lines here reported are preserved. Further directions of this work comprise the optimization of the biosensor, providing dimensional, material improvements, and acquisition frequency, to reach a two-fold or even three-fold sensitivity related to cellular dielectric properties changes. 

Indeed, technologies based on the use of biosensors are rapidly spreading in the field of clinical practice and medical diagnostics thanks to their unique features. In general, biosensors are simple and disposable, usually are cost-efficient and suitable for mass production, and are especially useful to discriminate between a healthy and pathological condition through a noninvasive approach. Many works in the literature have already demonstrated the efficacy and usefulness of biosensors in some human diseases with worldwide incidence and high morbidity and mortality, such as metabolic disorders (primarily diabetes), cardiovascular diseases, and cancer [52,53,54,55]. In addition, studies involving the use of microwave sensors have reported on their applicability in the medical field [10,49].

Our study, although it cannot yet be translated into clinical practice, provides evidence for a future investigation that should be based on primary cells from cancer biopsies. The strength of our work relies on the feasibility to discriminate between low-aggressive and metastatic cancer cells using only the measurement of dielectric cell properties. Indeed, most of the biosensors used in cancer prognosis are able to specifically recognize a cancer biomarker [56]. However, this could be a limitation with respect to those cancers in which specific biomarkers have been recognized in preclinical studies but are not widely used in medical practice, such as osteosarcoma [57]. Therefore, the future perspective of this study is to confirm its application as a prognostic tool when primary cells collected from human cancer biopsies are analyzed. To the best of our knowledge, this would be the first clinical application of a biosensor without the need for a specific molecular biomarker.

## 5. Conclusions

In this study, we proposed an innovative circular biosensor for detecting different adherent cancer cells based on their malignancy degree. First, the probe was designed and realized based on the shape and dimensions of a Petri dish and the operating frequency range of a network analyzer. Then, the sensor was used to evaluate the dielectric properties of four cancer cell lines deriving from different tissues of origin and with varying degrees of aggressiveness, and then compared with a culture medium. The experimental results clearly showed that the resonant frequency was the best parameter to assess differences in the dielectric properties of cell lines, as it turned out to be the most accurate and robust to cell variability and to potential confounding of external factors, such as the day of the experiment.

With regard to detecting the cells’ aggressiveness, the proposed sensor could discriminate between the medium alone and each tested cell line, and between low-aggressive versus high-aggressive cell lines, both within the same type of cancer and across the two types.

In conclusion, in this work, we suggested a novel resonator to discriminate different cell types, as well as the metastatic grade of cancer cells. This microwave approach offers a potential tool to support conventional diagnostic methods or in the long-term applications, particularly, for early cancer diagnosis. 

## Figures and Tables

**Table 1:** Mean ± SD of resonant frequency, *min|S_11_ (f)|,* full width at half maximum, and fitting error. *n* = 8.

Mean ± SD	Frequency (*f_r_*) [MHz]	Return Loss *(min|S_11_ (f)|)* [dB]	*FWHM* [MHz]	Fitting Error
DMEM	2230.92 ± 0.51	−13.87 ± 0.46	43.59 ± 2.87	303.01 ± 39.99
SaOS-2	2225.14 ± 0.48	−13.97 ± 0.84	45.11 ± 4.38	283.74 ± 64.02
143B	2223.24 ± 0.38	−14.29 ± 1.07	44.14 ± 4.12	271.96 ± 70.30
MCF7	2222.48 ± 0.22	−13.41 ± 0.45	46.89 ± 1.64	297.81 ± 35.06
MDA-MB-231	2223.81 ± 0.17	−13.37 ± 0.57	47.17 ± 1.67	271.42 ± 36.40

**Table 2:** MANOVA statistical results for comparing multiple variables jointly with respect to cell line and day factors.

Factors	Statistic	df	F	Prob > F
Cell line	0.0120	4	29.59	<0.0001
Days	0.4096	2	5.81	<0.0001

**Table 3:** ANOVA results for each dependent variable with respect to cell line and day factors.

	Resonant Frequency (*f_r_*)				Return Loss *min|S_11_ (f)|*				*FWHM*			
	*Coeff.*	*SE*	*t*	*p > |t|*	*Coeff.*	*SE*	*t*	*p > |t|*	*Coeff.*	*SE*	*t*	*p > |t|*
**Cell line**												
SaOS-2	–5.792	0.198	–29.190	<0.001	0.035	0.316	0.110	0.913	2.109	1.112	–0.154	0.067
143B	–7.611	0.196	–38.870	<0.001	–0.437	0.312	–1.400	0.171	0.264	1.098	–1.969	0.811
MCF-7	–8.461	0.210	–40.370	<0.001	0.054	0.334	0.160	0.874	0.091	1.175	–1.480	0.444
MDA-MB-231	–7.126	0.206	–34.580	<0.001	0.095	0.329	0.290	0.774	1.198	1.155	–1.152	0.307
**Days**												
D2	0.066	0.226	0.29	0.770	1.345	0.360	3.73	0.001	7.372	1.266	5.82	<0.001
D3	–0.008	0.168	–0.05	0.963	0.870	0.268	3.24	0.003	5.840	0.0943	6.19	<0.001

## Data Availability

Not applicable.
